# Short-Term Bacteriophage Exposure Is Associated with Shifts in Antibiotic Susceptibility Profiles of Clinical *Pseudomonas aeruginosa*

**DOI:** 10.3390/microorganisms14071585

**Published:** 2026-07-21

**Authors:** Nurullah Çiftçi, Özkan Şeşen, Güray Kor, Zeynep Özer, Uğur Vural, Zeynep Çelik, Mustafa Çilkız, İbrahim Halil Kılıç

**Affiliations:** 1 Department of Medical Microbiology, Faculty of Medicine, Kafkas University, Kars 36100, Türkiye; 2 Department of Biology, Faculty of Arts and Sciences, Gaziantep University, Gaziantep 27310, Türkiye; os111006@mail2.gantep.edu.tr (Ö.Ş.); uv211002@mail2.gantep.edu.tr (U.V.);; 3 Ankara Poultry Research Institute, Republic of Türkiye, Ministry of Agriculture and Forestry, Ankara 06170, Türkiye

**Keywords:** bacteriophage exposure, antimicrobial susceptibility testing, categorical susceptibility shifts, serial passage, β-lactam antibiotics, *Pseudomonas aeruginosa*

## Abstract

Bacteriophage exposure can impose strong selective pressure on bacterial populations and may alter antimicrobial susceptibility beyond direct lytic effects. However, the persistence of such changes after the removal of phage pressure remains insufficiently characterized in clinical *P. aeruginosa* isolates. This study evaluated whether short-term exposure to the lytic bacteriophage KPP10 was associated with changes in antimicrobial susceptibility categories and whether these changes remained detectable after serial passage in phage-free medium. Five non-duplicate clinical *P. aeruginosa* isolates were exposed to KPP10 for 24 h at a multiplicity of infection of 10. Antimicrobial susceptibility testing was performed by disk diffusion at baseline, immediately after exposure (F24) and after four serial passages in phage-free medium. Categories were interpreted according to the EUCAST 2024 clinical breakpoints. Nine categorical susceptibility shifts were detected across six antibiotics: four toward increased susceptibility and five toward decreased susceptibility. Six shifts (66.7%) remained detectable after four serial passages, whereas three shifts (33.3%), involving ciprofloxacin, cefepime, and aztreonam in LBK20, reverted to their baseline categories. Eight of the nine shifts involved β-lactam antibiotics, and cefepime was the most frequently affected agent. These exploratory findings show that short-term KPP10 exposure was associated with isolate-specific and bidirectional changes in categorical antimicrobial susceptibility and support repeated susceptibility monitoring in future phage–antibiotic studies.

## 1. Introduction

The global spread of multidrug-resistant (MDR) *P. aeruginosa* presents a major challenge in the management of healthcare-associated infections, particularly in settings where therapeutic options are limited. Resistance to antipseudomonal β-lactams may involve altered outer-membrane permeability, efflux regulation, chromosomal mechanisms, and acquired resistance determinants. These challenges have increased interest in complementary antibacterial strategies that may improve bacterial control or modify antimicrobial response profiles.

Bacteriophage therapy has re-emerged as a potential complement to conventional antibiotics, particularly against MDR Gram-negative pathogens such as *P. aeruginosa* [1,2,3]. Experimental studies have also demonstrated the therapeutic potential of lytic phages and phage cocktails against clinically relevant bacterial pathogens [4]. Clinical use has also been reported in difficult-to-treat *P. aeruginosa* infections [5]. In addition to direct lysis, phage exposure can impose selective pressure on bacterial populations and favor changes in surface structures involved in phage adsorption.

Phage-associated adaptation may alter antimicrobial susceptibility through evolutionary trade-offs. Selection for phage resistance has been linked to either increased or decreased susceptibility to particular antibiotics, depending on the phage receptor, bacterial genetic background, and antimicrobial class [6,7,8,9,10,11,12]. These bidirectional outcomes indicate that phage exposure should not be assumed to uniformly restore antibiotic susceptibility.

Some susceptibility changes may remain detectable after phage pressure is removed, whereas others may be transient [13,14]. Distinguishing persistent categorical shifts from reversible responses is relevant to the interpretation of post-phage antimicrobial susceptibility testing and to the design of future phage–antibiotic studies.

Population heterogeneity further complicates these interactions because pre-existing variants may be enriched during phage exposure [9,15,16,17]. However, conventional population-level antimicrobial susceptibility testing does not by itself distinguish population-wide change, clonal replacement, or the coexistence of phenotypically distinct subpopulations.

The present study therefore evaluated antimicrobial susceptibility categories in five clinical *P. aeruginosa* isolates at baseline, immediately after 24 h exposure to bacteriophage KPP10 and after four serial passages in phage-free medium. The objectives were to describe the direction, isolate specificity, and post-passage persistence of the observed categorical susceptibility shifts without assigning them to an untested molecular mechanism.

## 2. Materials and Methods

### 2.1. Bacterial Isolates

Five non-duplicate clinical *Pseudomonas aeruginosa* isolates (LBK7, LBK12, LBK20, LBK24, and LBK31) recovered from routine diagnostic specimens processed in a clinical microbiology laboratory in Kars, Türkiye, between 2024 and 2025 were included in the study. The isolates originated from urine (LBK7), a tracheal aspirate (LBK12), ear discharge (LBK20), a wound specimen (LBK24), and a tracheal aspirate (LBK31). They were recovered from five different patients, comprising four male patients and one female patient, with an age range of 47–83 years. Only one isolate per patient was included to prevent duplicate sampling. Species identification had previously been confirmed using conventional microbiological procedures and the VITEK^®^ 2 Compact automated identification system (bioMérieux, Marcy-l’Étoile, France). Baseline antimicrobial susceptibility testing was performed before bacteriophage exposure, and the study-specific baseline susceptibility profiles are presented in Table 1. Clonal relatedness among the isolates was not investigated. The isolates were stored at −80 °C in tryptic soy broth supplemented with glycerol until use.

### 2.2. Bacteriophage KPP10

The lytic bacteriophage KPP10, referred to as GAUN KPP10 in the doctoral thesis in which it was originally characterized, was previously isolated from an environmental water sample and characterized during doctoral research conducted at Gaziantep University [18]. In that work, KPP10 was identified as a lytic *P. aeruginosa* bacteriophage, and its adsorption characteristics, burst size, optimal pH, temperature stability, host interaction, and phage–antibiotic synergy with piperacillin/tazobactam against *P. aeruginosa* ATCC 27853 were evaluated [18]. The prior characterization has not yet been published as a peer-reviewed accession-linked report; therefore, the available information is cited here as thesis-based and laboratory-derived background for the reference phage and is interpreted cautiously.

Available laboratory-derived whole-genome sequence comparison and phylogenetic analysis placed KPP10 within a conserved lineage of Myovirus-like lytic *P. aeruginosa* bacteriophages. Genome similarity analysis was performed using Alfpy, followed by neighbor-joining tree construction and visualization with Interactive Tree of Life. Classification and annotation were additionally supported by BLAST comparisons with representative lytic *Pseudomonas* phages in GenBank using the NCBI BLAST web tool (https://blast.ncbi.nlm.nih.gov, accessed on 14 July 2026) and by analysis using the PhageScope database. No integrase-associated or other lysogeny-related markers were identified in the available genome annotation. However, because no validated public accession-linked genome record is currently available, genomic interpretation of KPP10 remains limited to the available thesis-based and laboratory-derived characterization data.

Previous host-range screening showed the lytic activity of KPP10 against the five clinical *P. aeruginosa* isolates included in this study. KPP10 has subsequently been maintained as a reference phage in our laboratory collection. Phage stocks were propagated on a susceptible *P. aeruginosa* host using the double-layer agar method, and phage concentrations were determined by plaque-forming unit enumeration before use.

### 2.3. Phage Propagation

Bacteriophage KPP10 stocks were propagated on a susceptible *P. aeruginosa* host using the standard double-layer agar plaque assay [19]. An overnight bacterial culture grown in Luria–Bertani broth at 37 °C with shaking was mixed with soft agar (0.7% agar) and poured over LB agar plates. Serially diluted phage suspensions were applied to the bacterial overlay and incubated at 37 °C for 18–24 h. High-titer lysates were prepared by flooding plates with sterile SM buffer, followed by gentle agitation and lysate recovery. The suspension was clarified by centrifugation at 10,000× *g* for 10 min using a UNIVERSAL 320 centrifuge (Andreas Hettich GmbH, Tuttlingen, Germany). The clarified lysate was passed through a sterile 0.22 µm cellulose acetate syringe filter (Sartorius, Göttingen, Germany) to remove residual bacterial cells and debris. Phage titers were determined by plaque-forming unit enumeration, and working suspensions were adjusted to the required concentration. Lysates were stored at 4 °C until use.

### 2.4. Host Range Confirmation Prior to Exposure Experiments

Before the exposure experiments, KPP10 activity against each clinical isolate was screened by spot testing. Bacterial lawns prepared from overnight cultures were overlaid with soft agar and challenged with high-titer phage suspensions. Plates were incubated at 37 °C for 18–24 h. Spot tests were used as preliminary screening, while productive infection was confirmed by plaque formation and efficiency-of-plating analysis. Only isolates showing productive infection were included in the exposure experiments.

### 2.5. Efficiency-of-Plating Analysis

The efficiency of plating (EOP) of KPP10 was determined for each clinical isolate using the standard double-layer agar method [19]. Serial tenfold phage dilutions were plated on each test isolate and on the reference propagation host. After incubation at 37 °C for 18–24 h, plaques were enumerated and EOP was calculated as the ratio of the titer on the test isolate to the titer on the reference host. Infectivity was classified as high (EOP ≥ 0.5), moderate (0.1 ≤ EOP < 0.5), low (0.001 ≤ EOP < 0.1), or inefficient (EOP < 0.001).

### 2.6. Growth Inhibition Kinetics (OD600 Monitoring)

The inhibitory effect of KPP10 on bacterial growth was evaluated by monitoring optical density at 600 nm (OD600) using a Multiskan™ GO microplate spectrophotometer (Thermo Fisher Scientific, Vantaa, Finland). Overnight cultures were diluted in fresh LB broth to an initial OD600 of approximately 0.05. KPP10 was added at a multiplicity of infection (MOI) of 10 [20], while parallel cultures without phage and medium-only wells served as controls. Cultures were incubated at 37 °C with orbital shaking using a Thermo Scientific™ MaxQ™ 4000 incubated orbital shaker (Thermo Fisher Scientific, Waltham, MA, USA). OD600 values were recorded at regular intervals during the 24 h incubation period. Each condition was measured in duplicate, and the growth curves were evaluated descriptively by comparing phage-exposed and untreated cultures.

### 2.7. Phage Exposure Experiment

Clinical *P. aeruginosa* isolates were exposed to KPP10 under controlled in vitro conditions. Overnight cultures were diluted in fresh LB broth to an initial OD600 of approximately 0.05, and KPP10 was added at an MOI of 10. Parallel cultures without phage served as untreated 24 h controls. All cultures were incubated for 24 h at 37 °C with orbital shaking at 100–150 rpm using a Thermo Scientific™ MaxQ™ 4000 incubated orbital shaker (Thermo Fisher Scientific, Waltham, MA, USA). After incubation, bacterial cells were recovered by centrifugation at 16,000 rpm for 5 min using a UNIVERSAL 320 centrifuge (Andreas Hettich GmbH, Tuttlingen, Germany) and resuspended in fresh LB broth. The antimicrobial susceptibility testing inoculum was prepared directly from the standardized surviving mixed-population suspension without selecting individual colonies. To assess whether post-exposure category changes remained detectable after the removal of phage pressure, phage-exposed cultures were subjected to four consecutive 24 h passages in fresh phage-free LB broth under the same incubation conditions (37 °C, orbital shaking at 100–150 rpm). Transfer into fresh medium at each passage reduced residual free-phage carryover before repeat antimicrobial susceptibility testing.

### 2.8. Antimicrobial Susceptibility Testing (AST)

Antimicrobial susceptibility testing (AST) was performed by disk diffusion according to the EUCAST clinical breakpoint tables, version 14.0 (2024) [21]. Overnight cultures of *Pseudomonas aeruginosa* isolates were adjusted to a turbidity equivalent to a 0.5 McFarland standard and inoculated onto Mueller–Hinton agar plates prepared using Mueller–Hinton agar (Merck KGaA, Darmstadt, Germany) with sterile cotton swabs. The antibiotic disks (Oxoid, Thermo Fisher Scientific, Basingstoke, UK) included ciprofloxacin (5 µg; CIP), cefepime (30 µg; FEP), ceftazidime (10 µg; CAZ), piperacillin–tazobactam (30/6 µg; TPZ), ceftazidime–avibactam (10/4 µg; CZA), and aztreonam (30 µg; ATM). The panel represented the prespecified antipseudomonal agents used for categorical comparison; the local prescribing frequency was not evaluated. Plates were incubated aerobically at 37 °C for 18–24 h, after which inhibition-zone diameters were measured and interpreted as susceptible (S); susceptible, increased exposure (I); or resistant (R). AST was performed at baseline, immediately after 24 h KPP10 exposure (F24) and after four serial passages in a phage-free medium. At each experimental stage, disk-diffusion AST was performed in triplicate under identical experimental conditions.

### 2.9. Definition of Susceptibility Shifts

Susceptibility categories were compared across baseline, F24, and Passage 4. A categorical susceptibility shift was defined as any change among S, I, and R after KPP10 exposure. Shifts toward increased susceptibility included R → I, R → S, and I → S; shifts toward decreased susceptibility included S → I, S → R, and I → R. A post-exposure category that remained unchanged at Passage 4 was termed a persistent categorical shift. A category that returned to its baseline interpretation after Passage 4 was termed a reverted shift.

### 2.10. Descriptive Assessment of Isolate-Specific Variability

Isolate-specific variability was described by comparing categorical trajectories at baseline, F24, and Passage 4. The study did not include single-colony susceptibility testing, population sequencing, transcriptomic analysis, or biochemical characterization of resistance mechanisms. Consequently, the observed trajectories were interpreted descriptively and were not assigned to a specific molecular or clonal mechanism.

### 2.11. Statistical Analysis

Categorical susceptibility transitions were analyzed descriptively for each isolate–antibiotic combination. The frequency, direction, and post-passage status of the shifts were summarized as counts and percentages. Disk-diffusion AST was performed in triplicate at baseline, F24, and Passage 4, whereas OD600 growth curves were evaluated descriptively using duplicate measurements. Because the study included five isolates and was designed as an exploratory phenotypic investigation, no inferential statistical testing was applied to the categorical AST outcomes. Data processing and graphical visualization were performed using GraphPad Prism software (version 8, GraphPad Software, Boston, MA, USA; https://www.graphpad.com/scientific-software/prism/, accessed on 14 July 2026)..

## 3. Results

### 3.1. Experimental Workflow of Susceptibility-Shift Analysis

**Figure 1:**
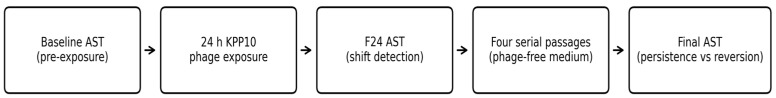
Experimental workflow used to evaluate categorical antibiotic susceptibility shifts following KPP10 exposure. Baseline AST was followed by 24 h phage exposure (F24), four serial passages in phage-free medium, and repeat AST.

Before susceptibility-shift analysis, KPP10 activity against all included clinical isolates was confirmed by host-range screening, plaque formation, EOP analysis, and OD600-based growth-inhibition experiments. Detailed isolate-specific OD600 growth curves are presented in Supplementary Figure S1. Each isolate was then evaluated at three stages: baseline, after 24 h KPP10 exposure (F24; MOI = 10), and after four serial passages in a phage-free medium. The experimental workflow of the susceptibility-shift analysis is summarized in Figure 1.

### 3.2. Baseline Antimicrobial Susceptibility Profiles of Clinical Isolates

Baseline AST demonstrated heterogeneous susceptibility profiles among the five clinical *P. aeruginosa* isolates. LBK7 was resistant to FEP, TPZ, ATM, and CZA, while remaining susceptible to CAZ and CIP. LBK20 showed reduced susceptibility to FEP, ATM, and CIP, and LBK24 showed reduced susceptibility to FEP and TPZ. LBK12 and LBK31 had broader baseline susceptibility profiles, with reduced susceptibility limited to CAZ and ATM, respectively. The baseline profiles are summarized in Table 1.

### 3.3. Antibiotic Susceptibility Shifts Following 24 h Phage Exposure

After 24 h KPP10 exposure, categorical susceptibility changes were detected in all five isolates. Nine shifts were identified across six antibiotics: four toward increased susceptibility and five toward decreased susceptibility. The changes predominantly involved β-lactam antibiotics, with FEP accounting for three of the nine shifts.

**Figure 2:**
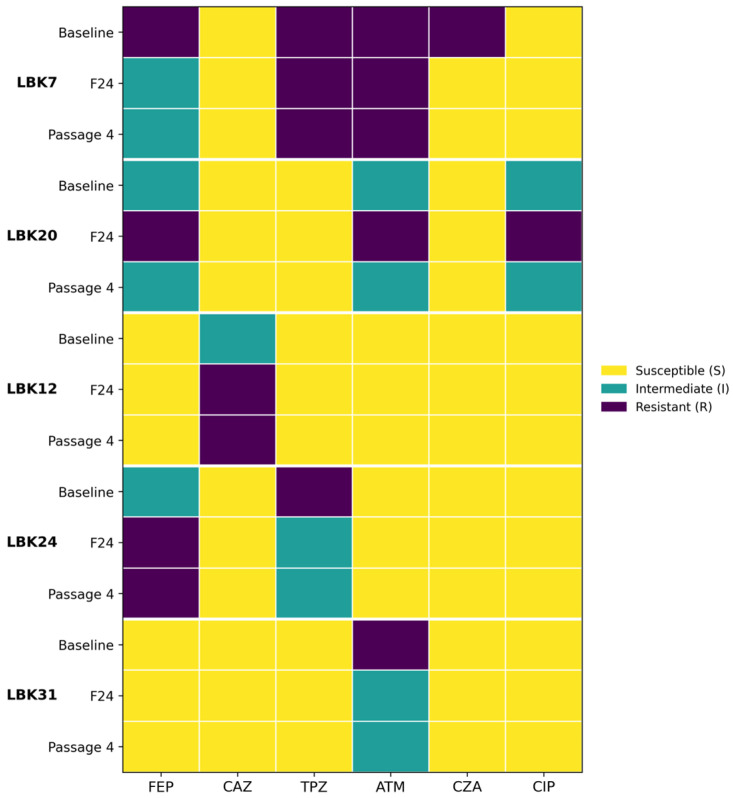
Heatmap showing antimicrobial susceptibility categories of clinical *P. aeruginosa* isolates at baseline, after 24 h KPP10 exposure (F24; MOI = 10) and after four serial passages in phage-free medium. Categories were interpreted according to EUCAST 2024 clinical breakpoints. Yellow indicates susceptible (S); teal indicates susceptible, increased exposure (I); and purple indicates resistant (R).

The distribution was isolate-specific. LBK20 exhibited three category transitions, LBK7 and LBK24 each exhibited two, and LBK12 and LBK31 each exhibited one (Figure 2 and Table 2). These observations demonstrate heterogeneous and bidirectional post-exposure susceptibility trajectories rather than a uniform change toward greater antibiotic susceptibility.

Representative Kirby–Bauer disk-diffusion images obtained at baseline and after KPP10 exposure are presented in Supplementary Figure S2.

### 3.4. Stability of Susceptibility Shifts After Serial Passages

After four serial passages in a phage-free medium, six of the nine post-exposure category shifts (66.7%) remained in the same category, whereas three shifts (33.3%) reverted to baseline. All three reverted shifts occurred in LBK20 and involved CIP, FEP, and ATM. Persistent shifts were observed in the remaining isolate–antibiotic combinations involving FEP, CAZ, TPZ, CZA, and ATM. Complete categorical trajectories are presented in Table 3 and the revised Supplementary Figure S3.

### 3.5. Antibiotic-Specific Distribution of Susceptibility Shifts

**Figure 3:**
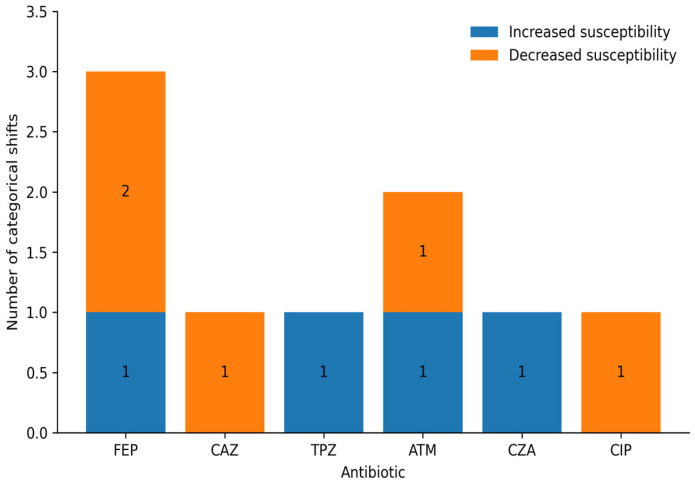
Antibiotic-specific distribution and direction of the nine categorical susceptibility shifts observed after 24 h KPP10 exposure. Stacked bars show shifts toward increased and decreased susceptibility. FEP, cefepime; CAZ, ceftazidime; TPZ, piperacillin–tazobactam; ATM, aztreonam; CZA, ceftazidime–avibactam; CIP, ciprofloxacin.

The nine shifts were not evenly distributed among the tested antibiotics. FEP accounted for three shifts, ATM for two, and CAZ, TPZ, CZA, and CIP for one shift each. Eight of the nine shifts involved β-lactam antibiotics, while one involved the fluoroquinolone CIP. The antibiotic-specific distribution and direction of the nine categorical susceptibility shifts observed after 24 h KPP10 exposure are shown in Figure 3. These counts are descriptive and should be interpreted in the context of the small isolate set. The antibiotic-specific distribution and post-passage status of the categorical susceptibility shifts are summarized in Table 4.

**Table 4:** Antibiotic-specific distribution of categorical susceptibility shifts and their status after serial passage.

Antibiotic	Shift Count	Persistent	Reverted
Cefepime (FEP)	3	2	1
Ceftazidime (CAZ)	1	1	0
Piperacillin–tazobactam (TPZ)	1	1	0
Aztreonam (ATM)	2	1	1
Ceftazidime–avibactam (CZA)	1	1	0
Ciprofloxacin (CIP)	1	0	1

Susceptibility categories were interpreted according to EUCAST 2024 clinical breakpoints.

### 3.6. Hypothesis-Generating Interpretation of Isolate-Specific Variability

**Figure 4:**
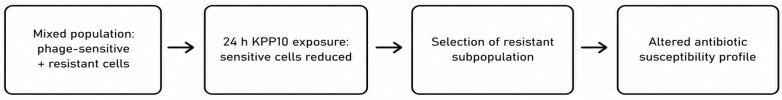
Hypothesis-generating model of one possible population-selection process following KPP10 exposure. The proposed enrichment of a pre-existing phage-resistant subpopulation was not directly demonstrated and required confirmation by colony-level phage susceptibility testing, quantitative AST, or genomic analysis.

LBK7 showed persistent category changes from R to I for FEP and from R to S for CZA. A possible explanation is the enrichment of a pre-existing subpopulation during phage exposure; however, this mechanism was not directly tested. The present population-level AST design cannot distinguish population-wide remodeling from clonal replacement or subpopulation enrichment. Figure 4 is therefore presented only as a hypothesis-generating conceptual model.

## 4. Discussion

Short-term KPP10 exposure was associated with nine categorical antimicrobial susceptibility shifts across all five clinical *P. aeruginosa* isolates. Four shifts were toward increased susceptibility and five were toward decreased susceptibility. Six shifts remained detectable after four serial passages, whereas three shifts in LBK20 reverted to baseline. Eight of the nine shifts involved β-lactam antibiotics. Together, these findings indicate bidirectional and isolate-specific post-exposure responses rather than the uniform restoration of antibiotic susceptibility.

Previous studies have shown that phage resistance or adaptation may involve changes in lipopolysaccharides, porins, outer-membrane proteins, and efflux-associated structures, with consequent effects on antimicrobial susceptibility [1,6,7,8,9,10,22]. The predominance of β-lactam-associated changes in the present dataset is phenotypically compatible with such trade-offs. Nevertheless, no receptor, permeability, efflux, β-lactamase, genomic, or transcriptional analysis was performed; therefore, these mechanisms remained hypotheses and the present findings did not establish antibiotic-class-specific causality.

Six of nine shifts (66.7%) remained in the same post-exposure category after four passages without added phage. This observation supports persistence at the level of categorical AST interpretation, but it should not be equated with genetically stable remodeling. A parallel untreated serial-passage control was not included, and the study did not quantify susceptibility distributions among individual colonies. Passage-associated variation, sampling effects, and changes in subpopulation proportions therefore cannot be excluded.

The LBK7 trajectory illustrates this interpretive limitation. Persistent changes were observed for FEP and CZA, and enrichment of a phage-resistant subpopulation was biologically plausible in light of the wider phage-resistance literature [15,23]. However, single-colony phage susceptibility, colony-level AST, and genomic comparison of baseline and post-exposure populations were not performed. The proposed population-selection model should consequently be regarded as hypothesis-generating rather than experimentally demonstrated.

The bidirectional nature of the shifts has practical implications for future phage–antibiotic investigations. KPP10 exposure did not uniformly improve antibiotic susceptibility; several isolate–antibiotic combinations instead shifted toward reduced susceptibility. Bidirectional antibiotic susceptibility effects following phage resistance have also been described in *Klebsiella pneumoniae*, supporting the broader relevance of this phenomenon beyond *P. aeruginosa* [12]. Experimental phage–antibiotic combinations have demonstrated context-dependent synergy in biofilm and infection models [22,24,25]. Antibiotic selection and timing in combination studies should therefore be guided by isolate-specific testing before and after phage exposure rather than by an assumption of universal collateral sensitivity. Direct phage–antibiotic combination or synergy experiments were not performed in this study, so no therapeutic interaction can be inferred from the present data.

Several limitations should be emphasized. The study included only five clinical isolates and one bacteriophage, limiting generalizability. Although KPP10 was previously isolated and characterized as GAUN KPP10 during doctoral research at Gaziantep University [18], this characterization has not yet been published as a peer-reviewed accession-linked report. Therefore, the absence of a publicly accessible genome accession, complete public genome annotation, and independently published biological characterization should be considered an important limitation. Genomic and mechanistic interpretations regarding KPP10 are consequently restricted to the available thesis-based and laboratory-derived data and should be interpreted cautiously. Clonal relatedness among the clinical isolates was not assessed. AST was based primarily on categorical disk-diffusion interpretations, and quantitative zone-diameter distributions and MIC confirmation were not systematically available for all isolate–antibiotic combinations. The post-exposure inoculum represented surviving mixed populations, without multiple-colony analysis. A parallel phage-free serial-passage control was absent. β-Lactamase production and resistance genes were not evaluated, and no genomic, transcriptomic, or biochemical mechanism was tested. In addition, the study did not directly evaluate phage–antibiotic combinations. The findings should therefore be interpreted as exploratory, phage-exposure-associated categorical susceptibility transitions.

Despite these limitations, the study indicates that susceptibility categories may change in either direction after short-term phage exposure and that some changes may remain detectable after phage removal. These observations support incorporating repeated, quantitative, and clone-aware susceptibility assessment into future studies of phage–antibiotic treatment strategies.

## 5. Conclusions

Short-term exposure to bacteriophage KPP10 was associated with isolate-specific and bidirectional categorical susceptibility shifts in clinical *P. aeruginosa*. Six of nine shifts remained detectable after four serial passages in a phage-free medium, while three reverted to baseline. Because the study was exploratory and lacked parallel serial-passage controls, clone-level analysis, and mechanistic validation, the findings did not establish a causal molecular pathway or therapeutic synergy. They nevertheless support monitoring antimicrobial susceptibility before and after phage exposure in future, larger phage–antibiotic studies.

## Figures and Tables

**Table 1:** Baseline antimicrobial susceptibility profiles of *P. aeruginosa* isolates.

Isolate	FEP	CAZ	TPZ	ATM	CZA	CIP
LBK7	R	S	R	R	R	S
LBK12	S	I	S	S	S	S
LBK20	I	S	S	I	S	I
LBK24	I	S	R	S	S	S
LBK31	S	S	S	R	S	S

Abbreviations: FEP, cefepime; CAZ, ceftazidime; TPZ, piperacillin–tazobactam; ATM, aztreonam; CZA, ceftazidime–avibactam; CIP, ciprofloxacin.

**Table 2:** Antibiotic susceptibility category shifts observed after 24 h exposure to bacteriophage KPP10 (MOI = 10) in clinical *P. aeruginosa* isolates.

Isolate	Antibiotic	Baseline	F24	Shift Direction
LBK20	CIP	I	R	Decreased susceptibility
LBK20	FEP	I	R	Decreased susceptibility
LBK7	FEP	R	I	Increased susceptibility
LBK24	FEP	I	R	Decreased susceptibility
LBK12	CAZ	I	R	Decreased susceptibility
LBK24	TPZ	R	I	Increased susceptibility
LBK7	CZA	R	S	Increased susceptibility
LBK31	ATM	R	I	Increased susceptibility
LBK20	ATM	I	R	Decreased susceptibility

Susceptibility categories were interpreted according to EUCAST 2024 clinical breakpoints. I denotes susceptible, increased exposure. Shift direction indicates movement toward increased susceptibility (R → I, R → S, or I → S) or decreased susceptibility (S → I, S → R, or I → R). Abbreviations: CIP, ciprofloxacin; FEP, cefepime; CAZ, ceftazidime; TPZ, piperacillin–tazobactam; CZA, ceftazidime–avibactam; ATM, aztreonam.

**Table 3:** Categorical antimicrobial susceptibility trajectories after KPP10 exposure and four serial passages in phage-free medium.

Isolate	Antibiotic	Baseline	F24	Passage 4	Status
LBK20	CIP	I	R	I	Reverted
LBK20	FEP	I	R	I	Reverted
LBK7	FEP	R	I	I	Persistent ↑
LBK24	FEP	I	R	R	Persistent ↓
LBK12	CAZ	I	R	R	Persistent ↓
LBK24	TPZ	R	I	I	Persistent ↑
LBK7	CZA	R	S	S	Persistent ↑
LBK31	ATM	R	I	I	Persistent ↑
LBK20	ATM	I	R	I	Reverted

Susceptibility categories were interpreted according to EUCAST 2024 clinical breakpoints. I denotes susceptible, increased exposure. In the Status column, ↑ and ↓ indicate persistent shifts toward increased and decreased susceptibility, respectively. Abbreviations: CIP, ciprofloxacin; FEP, cefepime; CAZ, ceftazidime; TPZ, piperacillin–tazobactam; CZA, ceftazidime–avibactam; ATM, aztreonam.

## Data Availability

The original contributions presented in this study are included in the article/Supplementary Materials. Further inquiries can be directed to the corresponding author.

## Supplementary Materials

The following supporting information can be downloaded at: https://www.mdpi.com/article/10.3390/microorganisms14071585/s1, Figure S1: OD600-based growth inhibition kinetics of clinical *Pseudomonas aeruginosa* isolates exposed to KPP10; Figure S2: Representative Kirby–Bauer disk-diffusion images before and after KPP10 exposure; Figure S3: Complete categorical antimicrobial susceptibility trajectories at baseline, after 24 h KPP10 exposure, and after four serial passages in phage-free medium.
