# Supplementary material for: Short-Term Bacteriophage Exposure Is Associated with Shifts in Antibiotic Susceptibility Profiles of Clinical Pseudomonas aeruginosa
Source: Microorganisms. 2026 Jul 21;14(7):1585. doi: 10.3390/microorganisms14071585 (PMC13414410; doi:10.3390/microorganisms14071585)

Short-term bacteriophage exposure is associated with shifts in antibiotic susceptibility profiles of clinical Pseudomonas aeruginosa

Supplementary Figure S1. OD600 Growth Curves

OD600 growth kinetics of clinical Pseudomonas aeruginosa isolates following bacteriophage KPP10 exposure at MOI 10 compared with untreated controls during 24 h incubation. Blue lines represent untreated bacterial controls, red lines represent phage-exposed bacterial cultures (MOI 10), and gray dashed lines represent medium-only controls. Shaded regions indicate replicate variability. Surviving phage-exposed bacterial populations obtained at 1440 min were subsequently used for antibiotic susceptibility shift analysis.

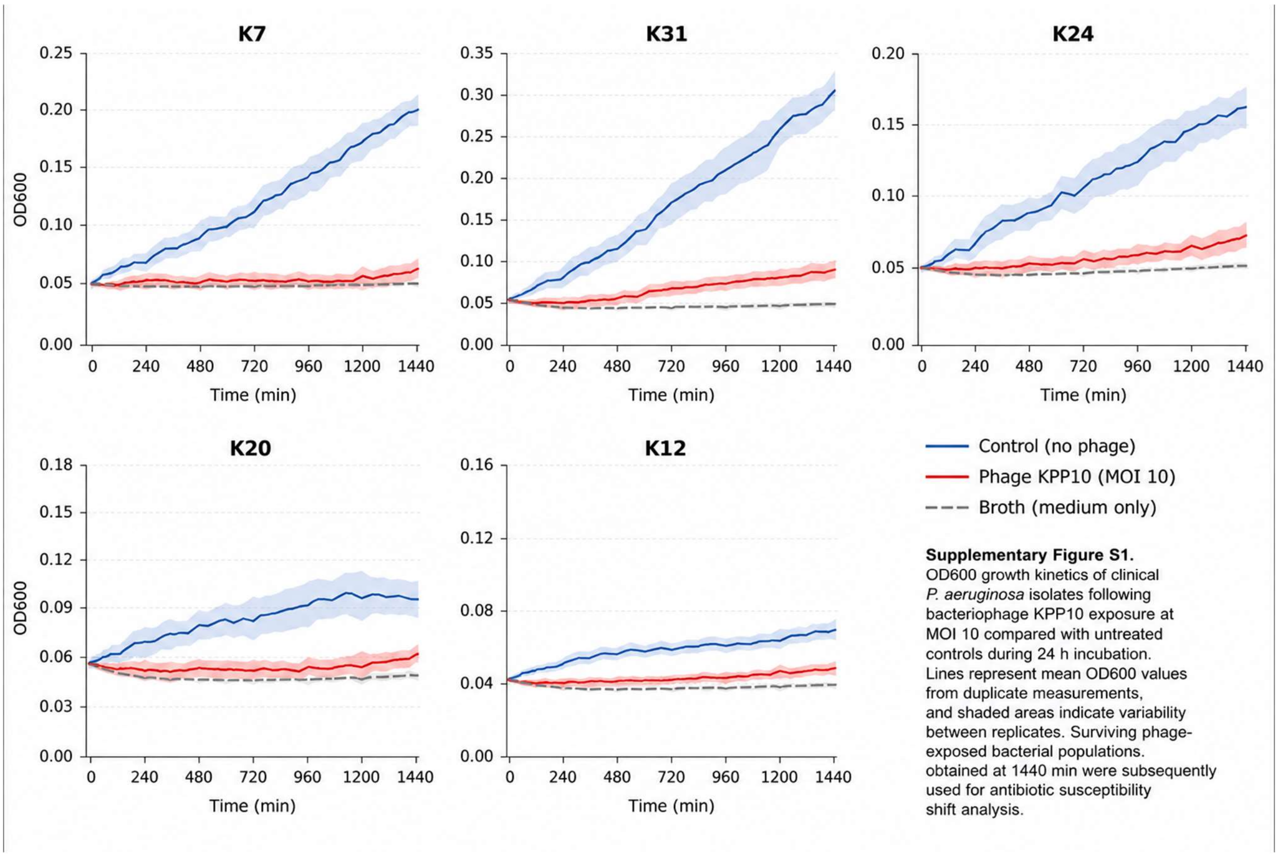

Supplementary Figure S2. Representative Before/After Disk Diffusion Plates

Representative Kirby–Bauer disk diffusion assay images demonstrating altered antimicrobial susceptibility profiles following 24 h bacteriophage KPP10 exposure (MOI = 10) in selected clinical Pseudomonas aeruginosa isolates. Paired baseline and post-phage exposure susceptibility profiles are shown for isolates exhibiting distinct phenotypic susceptibility transitions following bacteriophage exposure.

Supplementary Figure S2. Representative Before/After Disk Diffusion Plates

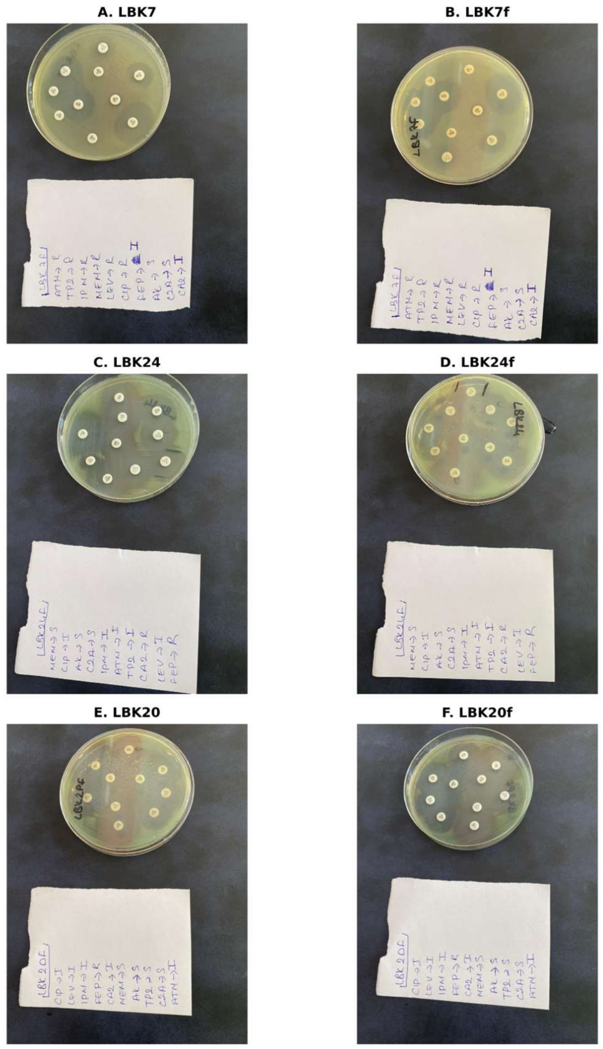

Supplementary Figure S3. Isolate-Specific Antimicrobial Susceptibility Trajectories

Heatmap-style matrix showing the categorical antimicrobial susceptibility trajectories of the five clinical Pseudomonas aeruginosa isolates at baseline, immediately after 24 h exposure to bacteriophage KPP10 (F24; MOI = 10), and after four consecutive serial passages in phage-free medium. Cell values are presented in the order Baseline → F24 → Passage 4. Gray cells indicate unchanged profiles, green cells indicate persistent shifts toward increased susceptibility, red cells indicate persistent shifts toward decreased susceptibility, and amber cells indicate transient shifts that returned to the baseline category after serial passage. S, susceptible; I, susceptible, increased exposure; R, resistant. Antibiotics: FEP, cefepime; CAZ, ceftazidime; TPZ, piperacillin-tazobactam; ATM, aztreonam; CZA, ceftazidime-avibactam; CIP, ciprofloxacin.

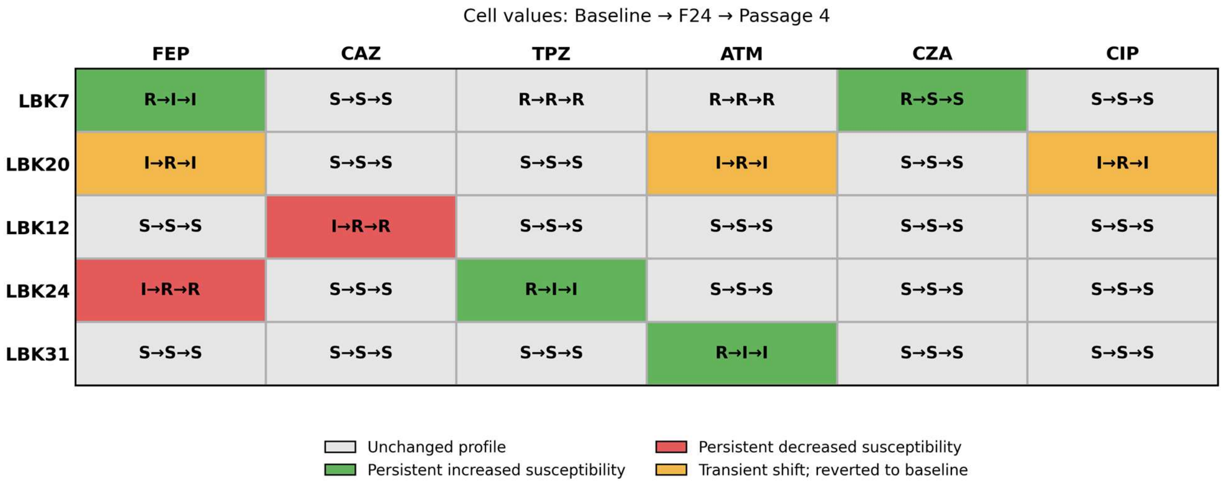

Supplement: Supplementary file 1 [file microorganisms-14-01585-s001.zip › microorganisms-4422512-supplementary.pdf]
